# Antifungal effects of BiOBr nanosheets carrying surfactant cetyltrimethylammonium bromide

**DOI:** 10.7555/JBR.32.20180043

**Published:** 2018-08-20

**Authors:** Mei-qing Sun, Zhan-lin Ding, Hong Wang, Guang-ping Yu, Li, Ming-chun Li, Meng-meng Zhen

**Affiliations:** 1. Key Laboratory of Systems Bioengineering (Ministry of Education), School of Chemical Engineering and Technology, Tianjin University, Tianjin 300072, China; 2. Wuqing District Center for Disease Control and Prevention, Tianjin 301700, China; 3. Ministry of Education Key Laboratory of Molecular Microbiology and Technology, College of Life Science, Nankai University, Tianjin 300071, China; 4. Tianjin Key Laboratory of Advanced Functional Porous Materials and Center for Electron Microscopy, Institute for New Energy Materials & Low-Carbon Technologies, School of Materials Science and Engineering, Tianjin University of Technology, Tianjin 300071, China.

**Keywords:** BiOBr nanosheets, *Candida albicans*, antifungal activity, cationic surfactant

## Abstract

BiOBr nanosheets are important photocatalytic nanomaterials. However, their biological effects remain to be explored. In this study, we investigated the antifungal effect of BiOBr nanosheets on *Candida albicans*. Strikingly, the nanosheets strongly inhibited the growth of *C. albicans* [IC_50_=(96±4.7) mg/L], hyphal development and biofilm formation. Compareed to the antifungal effect of the cationic surfactant cetyltrimethylammonium bromide, the inhibitory effect of the nanosheets on fungal pathogen was attributed to cetyltrimethylammonium bromide adsorbed by the nanosheets. Thermal gravity analysis and cetyltrimethylammonium bromide release experiment indicated that only 0.42% cetyltrimethylammonium bromide on BiOBr nanosheets was released. Taken together, this study uncovers the contribution of surfactant released from the nanosheets to their antifungal activity.

## Introduction

Nanomaterials have shown great promise in fields such as photocatalysis^[1–2]^, photothermal therapy^[3–4]^, biosensor^[5–6]^, diagnosis and imaging^[7–9]^. Nowadays, nanomaterials have attracted much attention in the research of anticancer drug carriers, photothermal therapy for cancer cells, etc., because of their intrinsic optical properties, including resonance Raman scattering, near-infrared (NIR) pho-toluminescence, and NIR optical absorption^[10]^.

BiOBr nanomaterials have high light-harvesting capacities under visible light irradiation. BiOBr can remove water pollutants, such as dye^[11–12]^, microcystin^[13]^, bisphenol^[14–15]^ and acetophenone^[16]^. Photocatalysis has already emerged as an advanced oxidation technology in water disinfection process^[5]^. However, the intensity of light decreases exponentially with water depth, significantly impacting on the effectiveness of disinfection. To our best knowledge, the biological effect of BiOBr nanomaterials in the dark remains to be investigated. On one hand, this biological effect is important for developing new multifunctional nanomaterials to improve disinfection systems in both light and dark conditions. On the other hand, it is desirable to prepare novel antimicrobial agents for clinical applications.

The antimicrobial activity of nanomaterials is usually tested using the representative pathogenic bacteria, such as *Escherichia coli* and *Staphylococcus aureus*^[17–18]^. These bacteria in drinking water and wastewater pose potential threat to public health and may cause various human infections. Pathogenic fungi are also pathogens. These eukaryotic organisms are implicated in life-threatening infections in both humans and animals. However, the knowledge on the antifungal effect of nanomaterials is very limited.

The aim of this work was to evaluate the effect of BiOBr nanosheets on pathogenic fungus and to explore the related cytotoxic mechanism. Compared to common cytotoxic mechanisms, such as the enhancement of vacuolar membrane permeabilization^[19]^, metal ion release^[20]^, cellular uptake^[21]^, and DNA damage^[22]^, the carried surfactant on the nanosheets contributed to the strong antifungal activity of the synthesized BiOBr nanosheets, which resulted in cell wall damage, reactive oxygen species (ROS) accumulation and mitochondrial dysfunction. This study implies that the BiOBr nanosheets are potential drug carriers of cationic surfactant for antifungal therapy.

## Materials and methods

### Strains

The *C. albicans* strain BWP17 (*ura3*Δ::λ*imm434/ura3*Δ::λ*imm434 his1*::*hisG/his1*::*hisG arg4*::*hisG/arg4*::*hisG*) used in this study was obtained from Prof. Dana Davis, University of Minnesota^[23]^. The strain NKF152 (*ura3*Δ::λ*imm434/ura3*Δ::λ*imm434 his1*::*hisG/his1*::*hisG arg4*::*hisG/arg4*::*hisG HWP1-GFP*) containing the Hwp1-GFP fragment, was used for Hwp1 localization. The strain was constructed by transforming the BWP17 with the hwp1::GFP::hwp1 fragment.

### Preparation and characterization of BiOBr nano-sheets

BiOBr nanosheets were prepared by a simple hydrothermal method. In a typical synthesis, 0.485 g of Bi(NO_3_)_3_·5H_2_O, 0.485 g of cetyltrimethylammonium bromide (CTAB, 99%, Aladdin , China), 20 mL ethylene glycol and 20 mL H_2_O were added to a 50 mL Teflonlined stainless autoclave. The suspension was stirred magnetically for 30 minutes to form a white precipitate. Then the teflonlined stainless autoclave was heated at 180 °C for 12 hours. After being cooled down to room temperature, the resulting precipitate was washed with distilled water and ethanol several times and dried at 60 °C for 6 hours, and thus the BiOBr nanosheets were obtained.

The general morphology of the products was characterized by field-emission scanning electron microscopy (FE-SEM, Nanosem 430, FEI) with an accelerating voltage of 0.1-30 kV and high-resolution transmission electron microscopy (HRTEM, Tecnai G^2^ F-20, FEI, USA). The crystal structure and composition of the sample were characterized by X-ray diffraction (XRD, D/max-2500, JAPAN SCIENCE). The surface groups were assessed by Fourier transformed infrared spectra (FT-IR, Bio-rad, FTS6000, USA) and thermal gravimetric (TG) was carried out on a thermoanalyzer in normal atmosphere (Rigaku Thermo plus EVO_2_ TG8121, Japan). The samples are heated in the temperature that increased at 10 °C/minute.

To obtain the stock solutions of BiOBr nanosheets, 10 mg of BiOBr nanosheets was suspended in 1 mL of YPD medium. The stock solution was vortexed for 30 minutes (AS3120, Autoscience, China) and subsequently diluted in YPD medium to obtain the appropriate concentrations.

To thoroughly remove the adsorbed surfactants on the BiOBr nanosheet surface, the nanosheets were suspended in 5% Tween 20 solution (prepared with distilled water), sonicated for 30 minutes (AS3120, Autoscience, China) and harvested by centrifugation. The pellets were further washed with 2.5% Tween 20 solution, 1% Tween 20 solution, distilled water, 50% ethanol (prepared with distilled water) and 100% ethanol. After being dried at room temperature, the nanosheets (which were named BiOBr-S) were further characterized by FT-IR and used for antifungal assays.

### Growth inhibition tests

To evaluate the inhibitory effect of BiOBr nanosheets on *C. albicans* growth, the wild-type (BWP17) cells were cultured overnight in YPD medium with shaking at 30 °C, adjusted to an initial optical density at 600 nm (OD_600_) of 0.1. BiOBr nanosheets were then added into the cell suspension with the following concentrations: 0, 0.63, 1.25, 2.50, 5.00, 10.00, 20.00, 40.00, 80.00, 160.00, 320.00, 640.00 mg/L. The suspensions were then incubated with shaking at 30 °C for 12 hours, and the cells were counted using haemocytometers. The percentage of growth (% growth) was calculated as the cell number in each group divided by that in the BiOBr nanosheet-free control × 100.

### Hyphal induction

*C. albicans* hyphae were induced in liquid RPMI-1640 medium. The overnight cultured cells of the strains BWP17 and NKF152 were harvested, washed twice with PBS, and diluted using RPMI-1640 medium to an OD_600_ of 0.1. BiOBr nanosheets were then added into the cell suspension with the concentrations described in the growth inhibition tests. The suspensions were cultured at 37 °C with shaking for 3 hours. Cells were centrifuged, fixed with 4% formaldehyde, and observed with a light microscope (Olympus, Japan). Both hyphal cells and total cells were counted, and the percentage of hyphal cells (% hyphal cells) was calculated as the number of hyphal cells divided by the number of total cells. At least twenty microscopic fields were detected.

### Biofilm formation assays

Biofilms were formed in 96-well microplates. Briefly, 50 μL RPMI-1640 medium containing BiOBr nanosheets ranging from 0.63 to 640 mg/L with 2-fold dilutions was added to the wells in the first eleven columns of the micortitre plates, and 50 μL RPMI-1640 medium without BiOBr was added to the wells in the last column as the control for biofilm formation. Then, 50 μL of *C. albicans* cell suspension (BWP17, 2×106 cells/mL) was added to each well. The plates were covered, sealed with parafilm and incubated at 37 °C for 24 hours. The medium was then removed, and the plate was washed 3 times with PBS. Metabolic activities of biofilms were assessed by the MTT (3-(4,5)-dimethylthiahiazo (-z-y1)-3,5-di-phenytetrazoliumromide) reduction assays. 100 μL of MTT solution (0.5 mg/mL, prepared in PBS, BBI, USA) was added to the washed wells. The plates were incubated in the dark at 37 °C for further 2 hours, and then the solution was removed from the wells. The plates were washed twice with PBS, and 100 μL of dimethyl sulfoxide (DMSO) was added into each well. The plates were gently shaken at room temperature for 5 minutes, and 90 μL of the extraction solution were aspirated into new microplates. The absorbance at 490 nm (OD_490_) in each well was determined using a microplate reader (Enspire, Perkinelmer, USA). For light microscopy, biofilm-cultured plates were washed with PBS 3 times, washed with distilled water to remove unadhered cells, and examined by a light microscope (Olympus, USA) with ×20 objectives for *C. albicans* morphology in the well bottom.

### Fluorescence microscopy

To visualize the cell wall and hyphal septa, the *C. albicans* cells (BWP17) were cultured in RPMI-1640 medium containing different concentrations of BiOBr nanosheets at 37 °C with shaking for 3 hours. Cells were then harvested, washed twice with PBS buffer, and stained with Calcofluor White (CFW, final concentration 10 mg/L, prepared in sterile water, Sigma) for 5 minutes. The stained cells were then observed by a fluorescence microscope (Olympus, Japan) with the 4',6-diamidino-2-phenylindole (DAPI) filter.

To measure the cell wall chitin content, the CFW-stained cells were collected, washed with PBS buffer and re-suspended in 1 mL fresh PBS buffer. The fluorescence density (FLU) of the cells was determined using a fluorescence microplate reader (excitation wavelength 325 nm, emission wavelength 435 nm, EnSpire, PerkinElmer, USA). For localization of the hyphal cell wall protein Hwp1, the strain NKF152 was cultured as described above. Cells were then harvested, fixed with 4% formaldehyde for 1 hour, and observed by the fluorescence microscope with the GFP filter.

### Statistical analysis

Each experiment was performed with three replicates in each treatment. The effect of BiOBr nanosheets on cell growth, hyphal development and biofilm formation was compared with the BiOBr nanosheet-free control. Student’s *t*-test was used to assess the significance of the difference (*P*<0.05) by the SPSS software (Version 20, IBM, USA).

## Results 

### Characterization of synthesized BiOBr nanosheets

**Fig.1:**
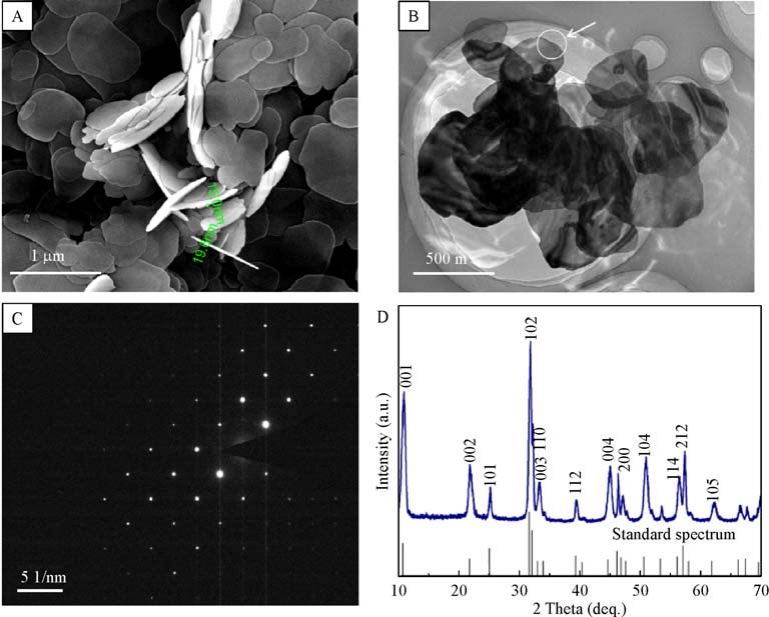
Characterization of the synthesized BiOBr nanosheets.

As shown in ***Fig. 1A***, the synthesized BiOBr consists of scale-like nanosheets with a size ranging from 0.5–1 μm and an average thickness of about 20 nm. The HRTEM image (***Fig. 1B***) and the selected area electron diffraction (SAED) spot pattern (***Fig. 1C***) show that BiOBr nanosheet is a single crystal and has a high crystallinity. XRD pattern of BiOBr nanosheets is depicted in ***Fig. 1D***, which is in good agreement with the standard spectrum of pure tetragonal phase BiOBr.

### BiOBr nanosheets inhibit ***C. albicans*** growth

**Fig.2:**
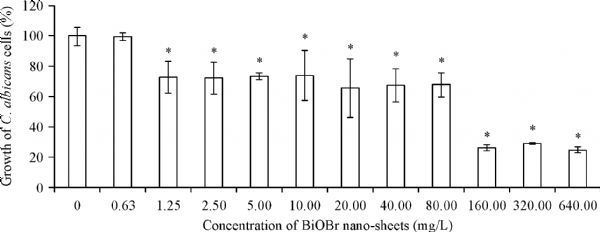
Effect of BiOBr nanosheets on growth of ***C. albicans***** cells. **

*C. albicans* poses great threat to human health, so developing strategies against this fungus has become a research focus. Evidence has showed that some kinds of nanomaterials have antifungal property. Here, we first tested the effect of BiOBr nanosheets on *C. albicans* growth. The inhibition growth assays demonstrated that the ≥2.5 mg/L of BiOBr nanosheets led to a significant decrease of growth biomass, and the biomass was reduced to 20%–30% when the nanosheet concentration reached 160 mg/L (***Fig. 2***). The IC_50_ of the nanosheets was (96±4.7) mg/L. Hence, the BiOBr nanosheets showed inhibitory effect on *C. albicans* growth.

### BiOBr nanosheets cause cell wall damage and ROS accumulation

**Fig.3:**
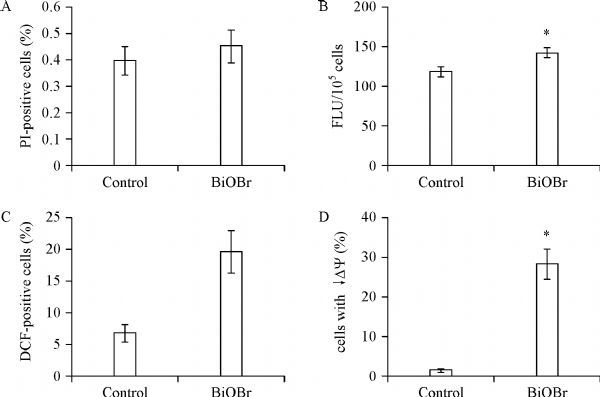
Effect of BiOBr nanosheets on plasma membrane damage (A), cell wall chitin contents (B), ROS accumulation (C) and mitochondrial membrane potential (κDY, D).

The toxicity of nanomaterials is usually attributed to plasma membrane damage, ROS-related oxidative stress or (and) dysfunction of macromolecules^[19]^. To explore the toxicity mechanisms of BiOBr nanosheets to *C. albicans*, plasma membrane damage was firstly assessed. Surprisingly, PI staining revealed that the nanosheets did not damage the fungal plasma membrane, since the PI-positive cells treated by the materials was<0.5%, having no significant difference from the control group (***Fig. 3A***).

Since the cell wall had direct interaction with the nanosheets, we further determined the effect of the nanosheets on the cell wall. Chitin, an essential component of the cell wall, was abundantly synthesized under cell wall stress^[24]^. CFW staining showed a significant increase of chitin content caused by BiOBr nanosheets (***Fig. 3B***). We also determined ROS contents and mitochondrial membrane potential (κDY) in the BiOBr-treated cells. It was demonstrated that the nanosheets induced ROS accumulation (***Fig. 3C***) and decreased κDY(***Fig. 3D***). Therefore, the inhibitory effect of BiOBr nanosheets was also associated with oxidative stress and mitochondrial dysfunction.

### BiOBr nanosheets inhibit ***C. albicans*** hyphal development

**Fig.4:**
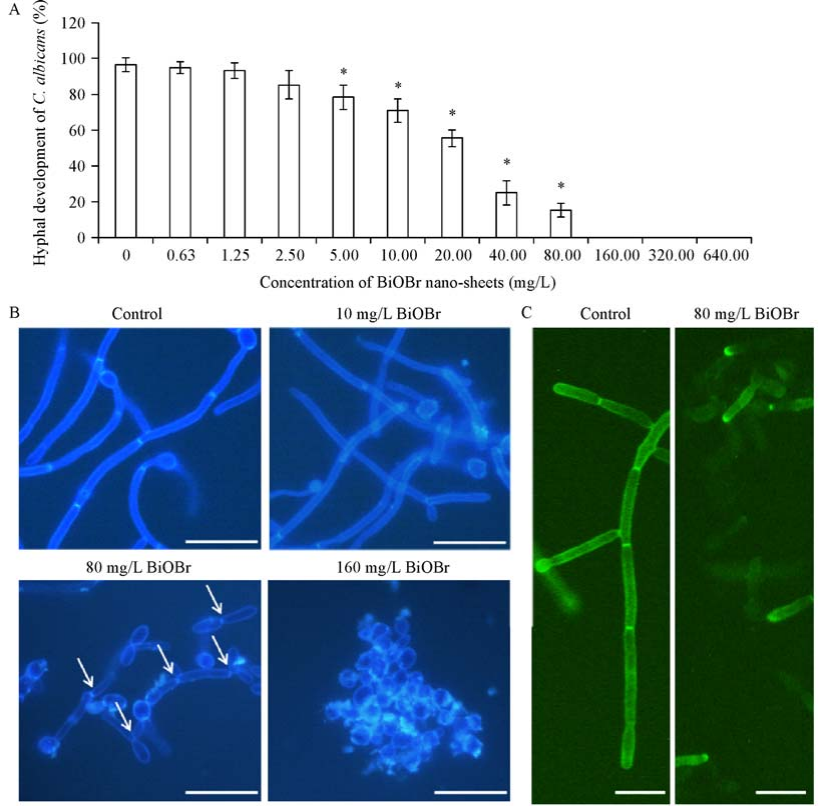
Effect of BiOBr nanosheets on hyphal development in ***C. albicans***.

Morphogenesis, such as hyphal development and biofilm formation, plays an essential role in the pathogenesis of *C. albicans*. Herein, we further investigated the effect of BiOBr nanosheets on morphogenesis of this pathogen. After 3 hours’ incubation in liquid RPMI-1640 medium, while>96% of the BiOBr-free cells produced regular true hyphae, ≥5 mg/L of BiOBr nanosheets caused a significant decrease of hyphal cells. Especially, 80 mg/L of nanosheets inhibited hyphal development in more than 80% cells, and ≥160 mg/L of the nanomaterials thoroughly blocked hyphal formation (***Fig. 4A***). CFW staining further demonstrated that BiOBr led to the production of hyphal branches at a low concentration (10 mg/mL) and a transition of true hyphae to pseudohyphae (80 mg/L, the pseudosepta, as an characteristic of pseudohyphae, indicated by white arrows) or yeast cells (160 mg/L) at high concentrations (***Fig. 4B***). The IC_50_ of the nanosheets to hyphal development was (29±3.2) mg/L. Moreover, Hwp1, a well-known hyphal cell wall protein, had much lower content in the BiOBr-treated cells than in the control cells, as indicated by the green fluorescence of Hwp1-GFP (***Fig. 4C***). These observations indicated the abnormal hyphal development caused by BiOBr nanosheets.

### BiOBr nanosheets inhibit ***C. albicans*** biofilm formation

**Fig.5:**
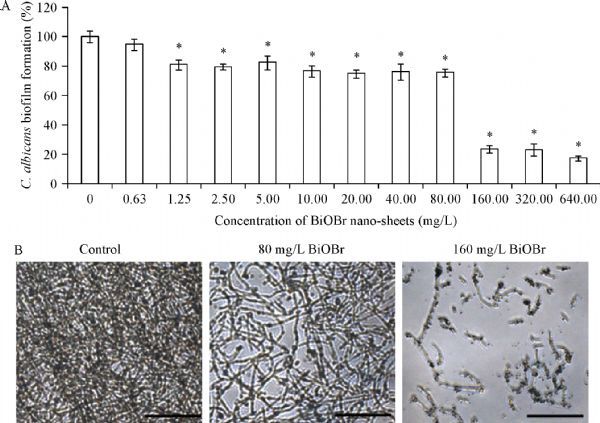
Effect of BiOBr nanosheets on ***C. albicans*** biofilm formation.

Biofilms play an important role in *C. albicans* resistance in various stress conditions and persistence in the host tissues. Biofilm formation is initiated by *C. albicans* adhesion to substrate surface and other fungal cells, which requires abundant adhesins, including Hwp1, and is closely associated with hyphal development^[25]^. Since the synthesized BiOBr nanosheets showed inhibitory effect on Hwp1 synthesis and hyphal development, we hypothesized that these materials might also inhibit biofilm formation in this pathogen. As expected, ≥1.25 mg/L BiOBr nanosheets significantly inhibited biofilm formation. Moreover, the biofilm formation was inhibited by approximately 80% when the BiOBr concentration reached 160 mg/L (***Fig. 5A***). The IC_50_ of the nanosheets to hyphal development was (126±5.8) mg/L. Microscopy further revealed that the nanosheets caused attenuation of biofilm formation (10 mg/L) and biofilm clearance (160 mg/L) (***Fig. 5B***). Taken together, these observations revealed the strong inhibition of the synthesized BiOBr nanosheets on *C. albicans* morphogenesis.

### The antifungal activity of BiOBr nanosheets is attributed to the adsorbed surfactant

**Fig.6:**
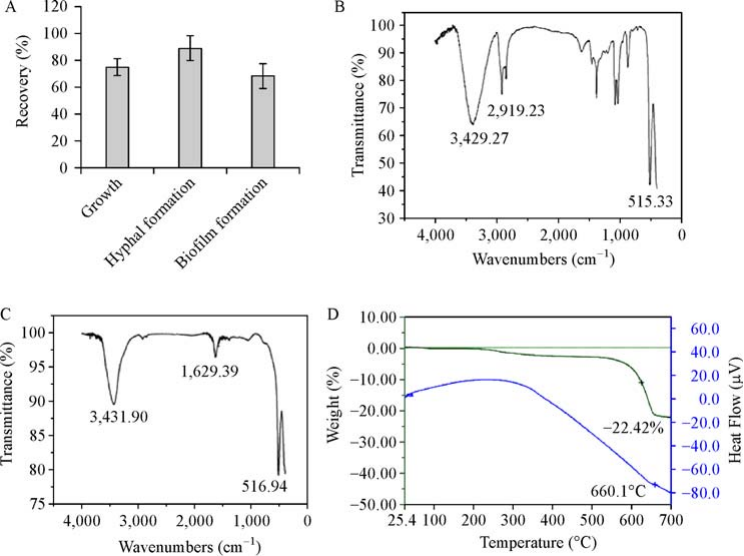
Toxicity caused by adsorption of cetyltrimethylammonium bromide (CTAB).

The antifungal effect of the treated nanosheets was further tested. Compared with untreated nanosheets, the BiOBr nanosheets without the surfactant (BiOBr-S), after wash with extra procedure had a 75.6% recovery in growth, an 89.8% recovery in hyphal development and a 70% recovery in biofilm formation (***Fig. 6A***). Hence, the adsorbed surfactants CTAB on the BiOBr nanosheets contributed to the toxicity of BiOBr nanosheets to the fungal cells. It has been reported that the ZnO-NPs in the presence of surfactants (i.e. SDS, CTAB) have antifungal activity^[26]^. It is also known that BiOBr was a good adsorbent for organic matter^[11]^, and it may adsorb the surfactant CTAB during synthesis. So we speculated that the toxicity of nanosheets could be attributed to CTAB. Interestingly, FT-IR measurement (***Fig. 6B***) clearly demonstrated that the symmetric and asymmetric-CH_2_- vibrations of CTAB appeared at 2,849 and 2,917 cm^−1^; moreover, the Bi-O stretching vibration peak appeared at around 515 cm^−1^ of BiOBr crystal, and the O-H stretching vibration of water appeared at (3,250–3,750) cm^−1^ respectively^[27–29]^. ***Fig. 6C*** showed that there were no obvious vibrations of CTAB at 2,849 and 2,917 cm^−1^ for BiOBr-S (***Fig. 6C***), indicating the thorough removal of the adsorbed surfactants on the nanosheets. In order to determine the content of CTAB adsorbed on the nanosheet surface, TG analysis was conducted. As observed in ***Fig. 6D***, the first decreased weight from 200 °C to 323.27 °C could be ascribed to the decomposition of the adsorbed CTAB^[30]^. Therefore, the value of the mass ratio of CTAB on BiOBr nanosheets was about 2.92%. Besides, the BiOBr was broken down when the temperature rose to 600 °C (***Fig. 6D***).

**Fig.7:**
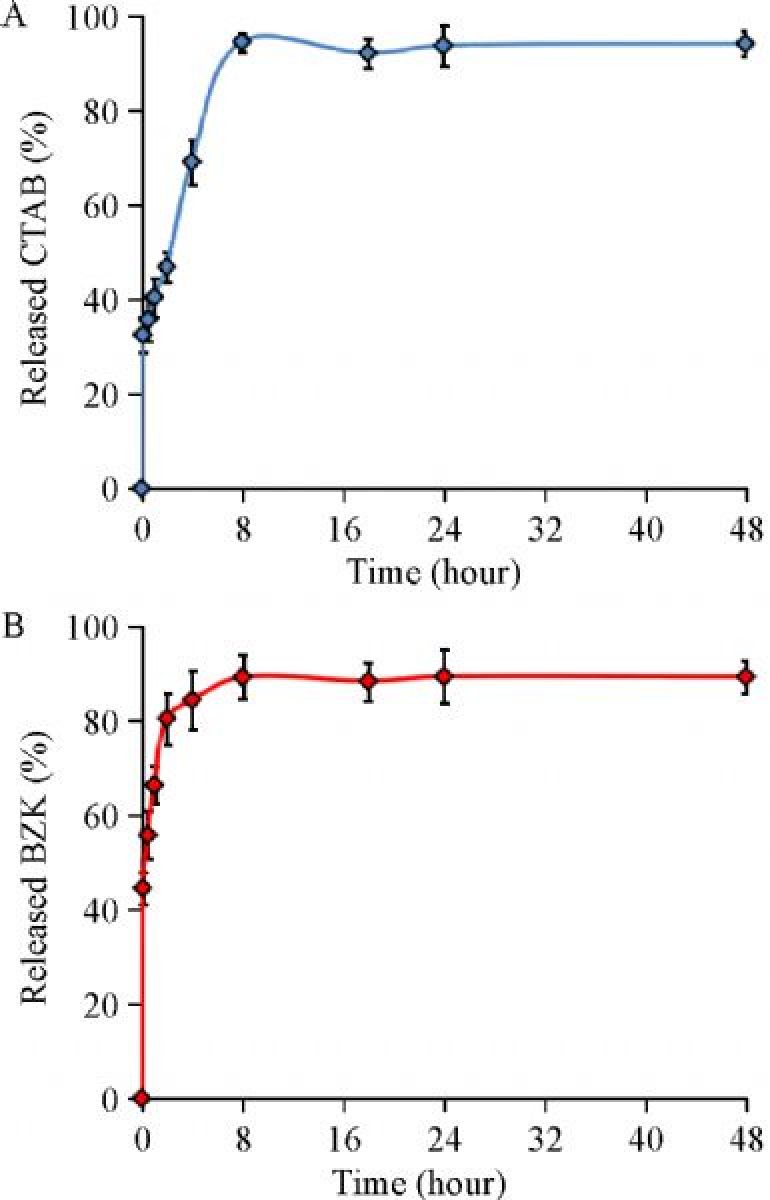
Sustained release capability of cationic surfactant.

Moreover, it has been reported that free CTAB exhibits stronger inhibitory effect on *C. albicans* growth [IC_50_: (0.75±0.14) mg/L], hyphal development [IC_50_: (0.18±0.03) mg/L], and biofilm formation [IC_50_: (0.88±0.12) mg/L]^[31]^, compared with the effect of CTAB carried by BiOBr on *C. albicans* growth [IC_50_: (1.92±0.094) mg/L], hyphal development [IC_50_: (0.58±0.064) mg/L], and biofilm formation [IC_50_: (2.52±0.116) mg/L]. Therefore, the CTAB adsorbed by BiOBr nanosheets showed lower toxicity than the same level of free CTAB, which may be attributed to the adsorption or hindering effect of BiOBr nanosheets. To prove this hypothesis, we did a CTAB release experiment to simulate drug release^[32–33]^. The method was shown in SI. It was shown that the CTAB release was relatively slow and sustained, and it took about 8 hours to reach equilibrium (***Fig. 7A***). At 48 hours, 94% CTAB was released (C_e_/C_i_, C_e_: the concentration of CTAB in equilibrium; C_i_: the concentration of CTAB initial). Based on a mass balance approach, 2.5% CTAB (weight _CTAB_/weight _BiOBr_) was still adsorbed. In TG analysis experiment, only 0.42% (weight _CTAB_/weight _BiOBr_) CTAB on BiOBr nanosheets in our synthetic materials was released. This result validates our hypothesis. Consistent with the result, BiOBr Nanosheets also exhibited 8 hours of slow and sustained release of another cationic surfactant, benzalkonium chloride, when it was made supersaturated (***Fig. 7B***). In conclusion, the BiOBr nanosheets showed great antifungal potentials as cationic surfactant drug carriers.

## Discussion

Although there is evidence indicating that BiOBr nanosheets are lowly toxic to various organisms under dark conditions^[11]^, herein we found that this nanomaterials had strong toxicity to the fungal model organism, *C. albicans*. Further investigations implied that this toxicity effect is attributed to the adsorbed surfactants on the surface of the nanosheets. Therefore, the adsorption activity of nanomaterials should be taken into account during investigation of the biological effect of nanomaterials. In this study, the synthesized BiOBr nanosheets had strong adsorption ability to the surfactants, which led to obvious toxicity to the tested fungus. Moreover, after releasing to the environment, nanomaterials (such as metal nanomaterials, metal oxide nanomaterials, carbon nanomaterials-graphene oxide, SWNTs, *etc*.) may further adsorb, migrate and transform of pollutants in the environment, the possible hazardous effect may be also involved in the toxicity of nanomaterials in the future.

This study for the first time investigates the biological effect of photocatalytic BiOBr nanosheets to pathogenic fungal organisms, and uncovers their excellent antifungal activity. This antifungal activity is attributed to the surfactants CTAB adsorbed by the nanosheets, rather than the direct plasma membrane damage caused by the nanosheets. This article enlightens that BioBr can not only be used in photocatalytic degradation but also as the cationic surfactant drug carriers in medicine. 
